# Tumor necrosis factor *α* decreases aquaporin 3 expression in intestinal epithelial cells through inhibition of constitutive transcription

**DOI:** 10.14814/phy2.13451

**Published:** 2017-10-16

**Authors:** Michael A. Peplowski, Andrew J. Vegso, Vadim Iablokov, Michael Dicay, Raza S. Zaheer, Bernard Renaux, David Proud, Morley D. Hollenberg, Paul L. Beck, Wallace K. MacNaughton

**Affiliations:** ^1^ Department of Physiology and Pharmacology University of Calgary Calgary Alberta Canada; ^2^ Department of Medicine University of Calgary Calgary Alberta Canada

**Keywords:** Aquaporin, inflammatory bowel disease, intestinal epithelium, transcription regulation, tumor necrosis factor (TNF)

## Abstract

Inflammatory diseases of the gut are associated with altered electrolyte and water transport, leading to the development of diarrhea. Epithelially expressed aquaporins (AQPs) are downregulated in inflammation, although the mechanisms involved are not known. We hypothesized that AQP3 expression in intestinal epithelial cells is altered in intestinal inflammation and that these changes are driven by tumor necrosis factor (TNF) *α*. Human colonic adenocarcinoma (HT‐29) cells were treated with TNF *α* to investigate signaling mechanisms in vitro. AQP3 expression was assessed by real‐time PCR and radiolabeled glycerol uptake, with select inhibitors and a luciferase reporter construct used to further elucidate intracellular signaling. AQP3 expression was downregulated in HT‐29 cells treated with TNF *α*. Luciferase reporter construct experiments revealed that TNF *α* downregulated constitutive transcriptional activity of the AQP3 promoter, and inhibition of MEK/ERK and nuclear factor *κ*B (NF‐*κ*B) signaling prevented the decrease in AQP3 mRNA expression. Constitutive AQP3 expression was suppressed by specificity protein (Sp) 3, and knockdown of this transcription factor bound to the AQP3 promoter was able to partially prevent the TNF *α*‐induced downregulation of AQP3. TNF *α* signals through MEK/ERK and NF‐*κ*B to enhance the negative transcriptional control of AQP3 expression exerted by Sp3. Similar mechanisms regulate numerous ion channels, suggesting a common mechanism by which both ion and water transport are altered in inflammation.

## Introduction

Aquaporins (AQPs) are a diverse family of channel proteins involved in the transmembrane passage of water and small solutes. AQP3 is highly expressed in the gastrointestinal tract and is localized to the basolateral membrane of intestinal epithelial cells (Ramirez‐Lorca et al. 1999; Silberstein et al. 1999; Rai et al. 2006). AQP3 belongs to a subclass of channels, known as the aquaglyceroporins, with permeability to water and other small solutes such as glycerol and urea (Ishibashi et al. 1994). AQP3 is particularly important for intestinal water transport, with inhibition or decreased expression of this channel resulting in diarrhea (Ikarashi et al. 2011, 2012), whereas increased AQP3 is characteristic of functional constipation (Yuan et al. 2008). This channel may also play a critical role in epithelial barrier integrity, with loss of AQP3 resulting in greater paracellular permeability (Zhang et al. 2011). Decreased AQP3 expression is also associated with gastrointestinal infections and may explain the development of diarrhea (Guttman et al. 2007). Reduced barrier integrity and diarrhea are similarly characteristic of inflammatory bowel diseases (IBD), which are also characterized by AQP downregulation (Hardin et al. 2004), including downregulation of AQP3 (Ricanek et al. 2015). The TNBS model of chemically induced colitis also shows decreased AQP3 expression (Zhao et al. 2014), although the molecular mechanisms driving this decreased expression have not been explored.

Tumor necrosis factor alpha (TNF*α*) is centrally involved in IBD, with several TNF*α* neutralizing antibodies (infliximab and adalimumab) being used therapeutically. TNF*α* is essential for the development of TNBS‐induced colitis (Neurath et al. 1997) and overexpression of TNF*α* results in the development of a Crohn's‐like ileitis in mice (Kontoyiannis et al. 1999). TNF*α* has also been shown to mediate the downregulation of ENaC, NHE_3_, and sodium potassium ATPase expression in colonic epithelial cells (Barmeyer et al. 2004; Markossian and Kreydiyyeh 2005; Amin et al. 2006). Although the regulatory mechanisms of AQP expression in the gut have not been characterized, it is known that TNF*α* is capable of both downregulating and upregulating AQP3 expression, depending on the cell type being studied, through distinct signaling pathways (Tancharoen et al. 2008; Horie et al. 2009). We sought to determine whether TNF*α* is involved in the downregulation of epithelial AQP3 and the mechanisms through which this downregulation is mediated. We show that TNF*α* reduces AQP3 expression in HT‐29 cells through MAP kinase and NF*κ*B pathways and by enhancing the suppressive actions of the transcription factor Sp3.

## Materials and Methods

### Cell culturing, cytokine treatment, and sample collection

HT‐29 human colorectal adenocarcinoma cells (American Type Culture Collection, Manassas, VA) were chosen due to the fact that their responses to inflammatory stimuli, such as TNF*α*, are similar to native colonic epithelia in terms of expression of cytokines and other inflammatory genes (Kolios et al. 1998). We have used this cell line, under the culture conditions described here, in previous studies of epithelial responses to TNF*α* (Iablokov et al. 2014). HT‐29 cells were cultured in DME/Ham's F‐12 media containing l‐glutamine (HyClone, Logan, UT), supplemented with 1% penicillin‐streptomycin solution (HyClone) and 10% fetal bovine serum (FBS) (Gibco, Grand Island, NY). Cells were routinely passaged before reaching confluence using 1.5X trypsin‐EDTA (Sigma‐Aldrich, Oakville, ON) and cell numbers quantified using a manual cytometer prior to plating. Once cells were confluent in 6‐ or 12‐well plates, cultures were treated with human recombinant TNF*α* (Cat#210‐TA, R&D Systems, Minneapolis, MN) for defined time periods and concentrations in the absence of serum. For AQP3 mRNA expression experiments equal to or longer than 24 h, media were supplemented with 1% FBS. For mRNA analysis, cells were collected and RNA purified using the RNeasy Mini Kit (Qiagen, Germantown, MD). Cells used for protein analysis were lysed in 200 *μ*L of buffer containing 50 mmol/L Tris‐HCl (pH 7.4), 10 mmol/L EDTA (EMD Chemicals), 1% Triton‐X‐100, and 2% Protease Inhibitor Cocktail (Sigma‐Aldrich) and collected on ice, with 20 mmol/L sodium fluoride (BDH) and 2 mmol/L sodium orthovanadate (Sigma‐Aldrich) added to the lysis buffer when assessing phosphoproteins.

### Reverse transcription and real‐time PCR

RNA samples isolated from cells were quantified using a NanoDrop 2000c spectrophotometer (Thermo Scientific, Wilmington, DE) and 700 ng were reverse transcribed using SuperScript II Reverse Transcriptase (Life Technologies, Carlsbad, CA) and random hexamer primers (GE Healthcare, Mississauga, ON). Real‐time PCR was performed using QuantiTect SYBR Green PCR mix (Qiagen) with previously published human *β*‐actin (100 nmol/L) (Watson et al. 1992) and AQP3 primers (300 nmol/L) (Okahira et al. 2008) synthesized in‐house (University of Calgary DNA Services). The Applied Biosystems 7900HT Fast Real‐time PCR System was used with three programmed steps running in standard mode: heat activation at 95°C for 10 min; 45 cycles of denaturation (95°C), primer annealing (57°C), and extension (72°C); and a dissociation curve. Data were collected using SDS2.3 and analyzed using the ΔΔCt method (Livak and Schmittgen 2001).

### Assessment of tritiated glycerol uptake

Uptake of radiolabeled glycerol to assess AQP3 function has been previously described (Ma et al. 1994, 2002; Yang and Verkman 1997; Jiang et al. 2011; Matsunaga et al. 2014). In order to assess the proportion of radiolabeled glycerol uptake mediated by AQP3, human AQP3 shRNA lentiviral particles (Cat#sc‐29713‐V, Santa Cruz, Dallas, TX) were used to stably transfect and downregulate AQP3 expression in HT‐29 cells. Scrambled shRNA control cells were transfected with Control (A) shRNA lentiviral particles (Cat#sc‐108080, Santa Cruz Biotechnology). Stably transfected HT‐29 cells expressing the shRNA constructs were selected using puromycin dihydrochloride (Santa Cruz Biotechnology) and serial dilutions in 96‐well plates were used to obtain single cell clones.

Untransduced HT‐29 and scrambled shRNA‐expressing cells were seeded onto 12‐well plates at a density of 5.0 × 10^5^ cells per well, whereas the less proliferative AQP3 shRNA‐expressing cells (M. A. Peplowski, unpublished data) were seeded at a starting density of 1.0 × 10^6^ cells per well and allowed to grow for 3 days to achieve similar cell counts at the end of the experiment. Two parallel sets of plates were prepared for use in tritiated glycerol uptake assessment and cell counting by manual cytometer. Untransduced, scrambled shRNA‐ and AQP3 shRNA‐expressing HT‐29 cells were placed in media lacking FBS for 1 h prior to TNF*α* (25 ng/mL) treatment for 24 h. Following treatment, duplicate wells of cells were incubated in DME/Ham's F‐12 media containing l‐glutamine supplemented with tritiated glycerol (1,2,3‐^3^H‐Glycerol, American Radiolabeled Chemicals Inc., St. Louis, MO) at a final concentration of 1 *μ*Ci/mL for 60 min. Cells were subsequently washed twice in PBS and lysed for 20 min in 300 *μ*L of buffer containing 50 mmol/L Tris‐HCl (pH7.4), 10 mM EDTA, and 1% Triton‐X‐100. Lysates were mixed with scintillation fluid, left overnight, and levels of beta emission were measured using a liquid scintillation counter (Beckman LS6500 Multipurpose Scintillation Counter).

### Treatment with signaling pathway inhibitors

HT‐29 cells were seeded onto 12‐well plates at a density of 5.0 × 10^5^ cells per well and grown for 3 days. Cells were placed in culture medium lacking FBS and containing inhibitor or vehicle control (DMSO; Sigma‐Aldrich) for 1 h prior to TNF*α* treatment, followed by addition and incubation with TNF*α* for an additional 12 h. To investigate signaling pathways downstream of TNF*α*, inhibitors of MEK (U0126, 10 *μ*mol/L, Promega, Madison, WI) and NF‐*κ*B (BAY11‐7082, 30 *μ*mol/L, Calbiochem, Etobicoke, ON) were used.

### Western blotting

Protein lysates collected for western blot were clarified by centrifugation at 20,800*g* for 10 min, followed by quantification using the detergent‐compatible protein assay (BioRad, Hercules, CA) with BSA as a standard. Samples were mixed with 5X loading dye (bromophenol blue, 5% SDS, 30% glycerol, 250 mmol/L Tris‐HCl pH 6.8), boiled for 10 min, resolved on 4–12% gradient SDS‐PAGE gels (BioRad), and transferred to nitrocellulose (BioRad). Blots were blocked, incubated overnight at 4°C in primary antibody as per the manufacturer's instructions (Sp1 antibody, Cat#9389, Cell Signaling, Beverly, MA; Sp3 antibody, Cat#sc‐644, Santa Cruz Biotechnology), incubated in secondary antibody (Anti‐rabbit HRP, Cat#111‐035‐144, Jackson ImmunoResearch, West Grove, PA) for 1 h at room temperature, and developed using Immobilon western chemiluminescent HRP substrate (Millipore, Etobicoke, ON). Images of western blots were captured using QuantityOne software on the BioRad ChemiDoc XRS System and quantified by densitometry.

### siRNA transfection and transient knockdown

Subconfluent HT‐29 cells were passaged and washed twice in antibiotic‐free media, followed by seeding at a density of 1.0 × 10^6^ cells per well in a 12‐well plate. Immediately following seeding, 200 *μ*L of premixed OptiMEM Media (Gibco) containing siRNA and 20 *μ*L of Lipofectamine RNAiMAX reagent (Life Technologies) were added dropwise to each well, resulting in final siRNA concentrations of 100–300 nmol/L (Sp1 siRNA, Cat#sc‐29487; Sp3 siRNA, Cat# sc‐29490, Santa Cruz Biotechnology; AllStars Negative Control siRNA, Cat#1027281, Qiagen). Cells were transiently transfected in suspension and allowed to adhere to the base of the well overnight, followed by switch to FBS‐free media for 1 h and treatment with cytokine for 12 h in FBS‐free media. Cells were washed twice in PBS and collected for mRNA or protein analysis.

### Promoter construct generation, truncations, and site‐directed mutagenesis

Genomic DNA was isolated from HT‐29 cells using the QIAamp DNA Mini Kit (Qiagen). AQP3 promoter fragment −1680 to −74 was amplified using custom synthesized primers (Table 1; University of Calgary DNA Services) and Herculase II Fusion DNA Polymerase (Agilent Technologies, Santa Clara, CA). The pGL4.10[luc2] vector (Promega, Madison, WI) and promoter fragment were digested with the restriction enzymes, KpnI and NheI (New England BioLabs, Ipswich, MA), to allow for directional cloning. To prevent self‐ligation, pGL4.10[luc2] vector was treated with antarctic phosphatase (New England BioLabs, Ipswich, MA). The vector and promoter fragments were ligated using Rapid DNA Ligation Kit (Roche, Mannheim, Germany) and transformed into One Shot TOP10 Chemically Competent *E. coli* (Life Technologies). Insert identity was confirmed by sequencing (University of Calgary DNA Services). Truncation constructs were generated using different forward primers (Table 1) and cloned into pGL4.10[luc2] as described above, whereas the ‐95 construct was generated by reverse amplifying the full construct backbone, KpnI digestion, ligation, and transformation of the product. Sp1 site‐directed mutant (SDM) construct promoters were generated using mutagenesis by overlap extension (Heckman and Pease 2007), followed by cloning into pGL4.10[luc2] vector. They were screened using restriction digestion (Sp1 SDM in the presence of BamH1), and insert identity was confirmed by sequencing (University of Calgary DNA Services). The Plasmid Maxi Kit (Qiagen) was used to isolate sufficient quantities of plasmid DNA for transfection experiments.

**Table 1:** Primer sequences used to generate the AQP3 promoter constructs, truncations, and site‐directed mutants

Primer Name	Sequence
Promoter Reverse	AAAAAAGCTAGCAGCGCTGGTGGCTCCCTTTATA
−1680 Forward	AAAAAAGGTACCGCCACCAGATGTTTCCTTGTTA
−966 Forward	AAAAAAGGTACCTGGGAGAGGAGAGGGTTAAAA
−761 Forward	AAAAAAGGTACCAGGACATCCGCCATGTGTAG
−346 Forward	AAAAAAGGTACCTAATCCCACCATTGGCTCTC
−166 Forward	AAAAAAGGTACCCGCACTCCTCGGCGCTCC
−95 Forward	AAAAAAGGTACCTATAAAGGGAGCCACCAGCGC
−95 Reverse	AAAAAAGGTACCGGCCAGTTAGGCC
Sp1 SDM Forward	CTCCCAGCCGAGGTGGATCGGGATCCAGGGATCGCGCACTCCTCGGCGCTC
Sp1 SDM Reverse	GGAGTGCGCGATCCCTGGATCCCGATCCACCTCGGCTGGGAGGC

### Luciferase assay

The AQP3 promoter constructs generated above were used for elucidation of AQP3 promoter elements involved in the cytokine‐induced decrease in AQP3 expression. Cells were passaged and washed twice in antibiotic‐free media, followed by seeding at a density of 2.0 × 10^6^ cells per well in a 12‐well plate. Immediately following seeding, 100 *μ*L of premixed OptiMEM Media (Gibco, Grand Island, NY) containing 2 *μ*g of plasmid DNA and 6 *μ*L of TransIT‐LT1 transfection agent (Mirus Bio, Madison, WI) were added dropwise to each well. Cells were allowed to adhere to the base of the well for 5 h, followed by switch to serum‐free media for 1 h and subsequent treatment with cytokine for 12 h in serum‐free media. Cells were washed twice in PBS and collected by scraping in 250 *μ*L of 1X lysis buffer supplied with the Firefly luciferase assay kit (Biotium, Hayward, CA). Twenty microliter of lysate was added per reaction and luminosity generated from D‐luciferin substrate (Biotium) was assessed over a 10 sec period. Data were normalized to the untreated, full AQP3 promoter construct (−1680).

### Chromatin Immunoprecipitation

Chromatin immunoprecipitation (ChIP) was performed using the SimpleChIP Enzymatic Chromatin IP Kit with Agarose Beads (Cell Signaling Technology). HT‐29 cells were seeded onto 100‐mm‐diameter dishes at a density of 7.5 × 10^6^ cells per dish and allowed to grow for 3 days to reach ~4.0 × 10^7^ cells per dish. Cells were placed in culture medium lacking FBS for 1 h prior to treatment with TNF*α* (25 ng/mL) for 4 h. Following cross‐linking, isolated nuclei were digested with 5 *μ*L micrococcal nuclease for 20 min. Chromatin fragments were immunoprecipitated overnight with Sp3 (5 *μ*g) or Histone H3 (2.78 *μ*g) antibodies or an equivalent amount of rabbit IgG isotype antibody, followed by pull down using agarose beads for 2 h. Isolated DNA fragments were amplified by PCR using primers for the AQP3 promoter (Forward 5′–GAAGTGGGGTGATCACAGGG, Reverse 5′–TCTGAGAGCCAATGGTGGGA, 60°C annealing temperature) or Ribosomal Protein L30 (RPL30, Cat#7014, Cell Signaling) promoter. PCR products were run on a 2% agarose gel containing 0.1 *μ*g/mL ethidium bromide and images were captured under UV light using QuantityOne software on the BioRad ChemiDoc XRS System.

### Statistical analysis

All groups analyzed statistically had sample sizes of at least three and are plotted as the mean ± SEM. For analyses in experiments where there were only two groups, a Student's unpaired *t*‐test was performed. When three or more groups were present, an analysis of variance (ANOVA) was performed with either a Newman–Keuls post hoc test (up to four groups being compared) or a Tukey's post hoc test (greater than four groups being compared). A *P* < 0.05 was considered statistically significant.

## Results

### TNFα downregulates AQP3 mRNA expression in HT29 cells

**Figure 1:**
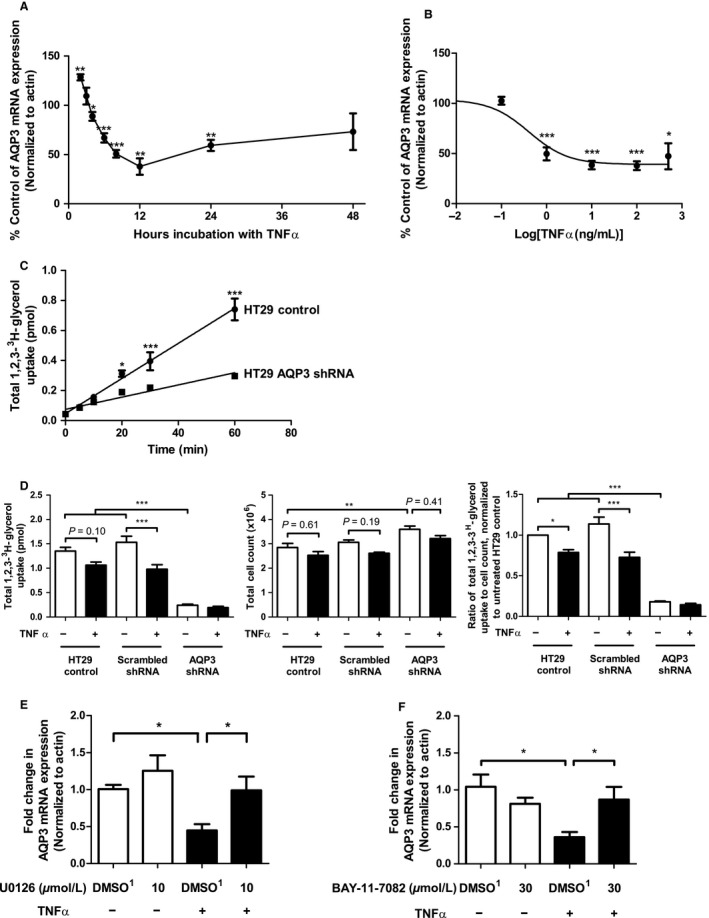
AQP3 mRNA and protein expression are decreased following treatment with TNF *α* and this effect can be prevented by pretreatment with inhibitors of MEK/ERK and NF‐*κ*B signaling pathways. AQP3 mRNA expression was assessed by real‐time RT‐PCR in HT‐29 cells treated with TNF *α* (25 ng/mL) for the times indicated (A) or for a total of 12 h in a concentration‐dependent fashion as indicated (B). Statistical significance was assessed in comparison to untreated samples using an unpaired *t*‐test (A) or ANOVA (B) (*P < 0.05, **P < 0.01, ***P < 0.001). *n* = 3–4. A time course experiment was performed to assess 1,2,3‐^3^H‐glycerol uptake into untransfected and AQP3 shRNA‐transfected HT‐29 cells and demonstrated significantly decreased uptake at the 20, 30, and 60 min time points (*n* = 4, *P < 0.05, ***P < 0.001) (C). Uptake of 1,2,3‐^3^H‐glycerol was assessed in untransfected, scrambled shRNA and AQP3 shRNA‐expressing HT‐29 cells under control and TNF *α* (25 ng/mL)‐treated conditions (D). Total cellular ^3^H uptake was assessed using liquid scintillation counting (D, left panel), cell counts were performed by manual hemacytometer on a parallel set of cells (D, middle panel) and data were normalized to determine the total ^3^H uptake per cell (D, right panel). Statistical significance was assessed by ANOVA and a Tukey's post hoc test (*n* = 6; *P < 0.05; **P < 0.01; ***P < 0.001). MAPK inhibitor U0126 significantly abrogated the TNF *α*‐induced downregulation of AQP3 mRNA expression at the 12 h time point as shown by real‐time RT‐PCR (E), with significance assessed by ANOVA and a Newman–Keuls post hoc test (lower right; *n* = 5; *P < 0.05). TNF *α*, 25 ng/mL; DMSO ^1^, Volume of DMSO equivalent to 10 *μ*mol/L U0126. NF‐*κ*B inhibitor BAY‐11‐7082 abrogated the TNF *α*‐induced downregulation of AQP3 mRNA expression at the 12 h time point as shown by real‐time RT‐PCR (F), with significance assessed by ANOVA and a Newman–Keuls post hoc test (lower right; *n* = 4–5). TNF *α*, 25 ng/mL; DMSO ^1^, Volume of DMSO equivalent to 30 *μ*mol/L BAY‐11‐7082.

TNF*α* (25 ng/mL) significantly increased AQP3 expression at 2 h, followed by significantly decreased AQP3 expression at the 4, 6, 8, 12, and 24 h time points postaddition of cytokine (Fig. 1A). Similarly, TNF*α* treatment for 12 h significantly decreased AQP3 mRNA expression at concentrations of 1, 10, 25, and 100 ng/mL, with an IC_50_ of 0.41 ng/mL (Fig. 1B).

We experienced significant challenges in detecting AQP3 protein expression in HT29 cells using all commercially available antibodies (data not shown). To circumvent these technical challenges, we measured the uptake of radiolabeled 1,2,3‐^3^H‐glycerol which has previously been used by others to assess functional changes in AQP3 membrane expression (Jiang et al. 2011; Matsunaga et al. 2014) that would be expected to correlate with AQP3 protein levels. We observed a time‐dependent linear increase in 1,2,3‐^3^H‐glycerol uptake that was significantly dampened in cells expressing AQP3 shRNA, suggesting that AQP3 protein was expressed in HT‐29 cells and its downregulation could be assessed using this approach (Fig. 1C).

Cognizant of the alteration in proliferative capacity of AQP3 knockdown cells, coupled with the apoptotic‐induction potential of cytokines, we assessed tritiated glycerol uptake normalized to cell number in untransfected, scrambled shRNA and AQP3 shRNA‐expressing HT‐29 cells treated with TNF*α*. Overall, when tritiated glycerol levels were taken as a ratio of cell counts on a parallel plate and normalized to untreated HT‐29 control cells, we found that TNF*α* significantly reduced tritiated glycerol levels in cellular lysates of untransfected (HT‐29) and scrambled shRNA‐treated cells, but not in AQP3 shRNA‐expressing cells (Fig. 1D). TNF*α* also did not significantly reduce cell counts (Fig. 1D).

### Prevention of TNFα‐induced pERK and NF‐κB signaling abrogates the TNFα‐induced decrease in aquaporin 3 mRNA expression

The TNF*α*‐mediated decrease in AQP3 mRNA expression was prevented by the MEK/ERK signaling inhibitor U0126 (Fig. 1E), implicating MEK as an upstream kinase in the signaling pathway responsible for the TNF*α*‐induced decrease in AQP3 mRNA expression. U0126 did not significantly alter constitutive AQP3 mRNA expression in HT‐29 cells treated with inhibitor alone.

Activation of NF‐*κ*B signaling by TNF*α* is a well‐characterized constitutive signaling pathway. Cells pretreated with the IKK inhibitor that blocks NF‐*κ*B activation (BAY‐11‐7082) prevented the TNF*α*‐induced downregulation of AQP3 mRNA expression compared to the DMSO vehicle/TNF*α*‐treated control (Fig. 1F), suggesting that IKK‐mediated NF‐*κ*B activation is involved in the downregulation of AQP3 mRNA expression driven by TNF*α*.

### TNFα decreases aquaporin 3 mRNA expression through inhibition of constitutive transcription

**Figure 2:**
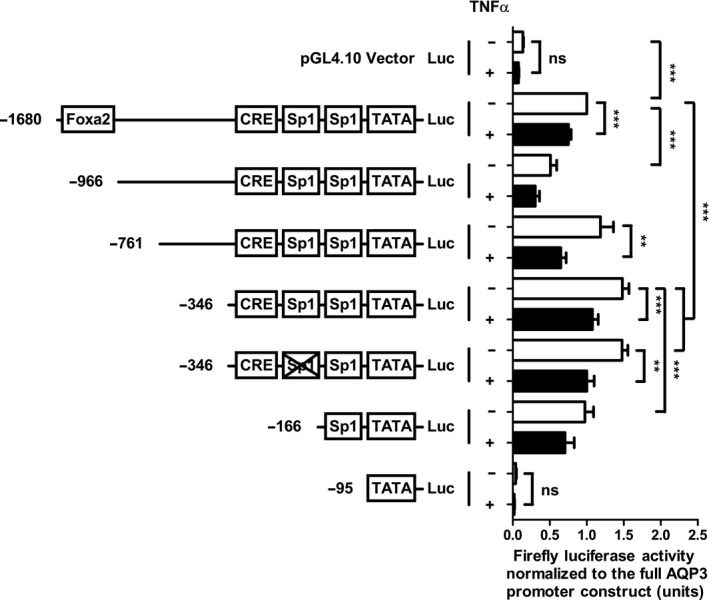
Proximal AQP3 promoter elements are sufficient to drive constitutive expression and mediate the TNF *α*‐induced downregulation of AQP3 mRNA expression. HT‐29 cells were transiently transfected with the full AQP3 promoter construct (−1680), truncations (−966, −761, −346, −166, and −95), or a site‐directed mutant (−346 lacking Sp1) cloned into the pGL4.10 firefly luciferase vector for assessment of promoter activity and response to TNF *α* (25 ng/mL for 12 h). Lysates were assessed for luminescence generated in the presence of D‐luciferin and normalized to the full AQP3 promoter construct. Statistical significance was assessed by ANOVA and a Tukey's post hoc test (*n* = 5–28; **P < 0.01; ***P < 0.001).

**Figure 3:**
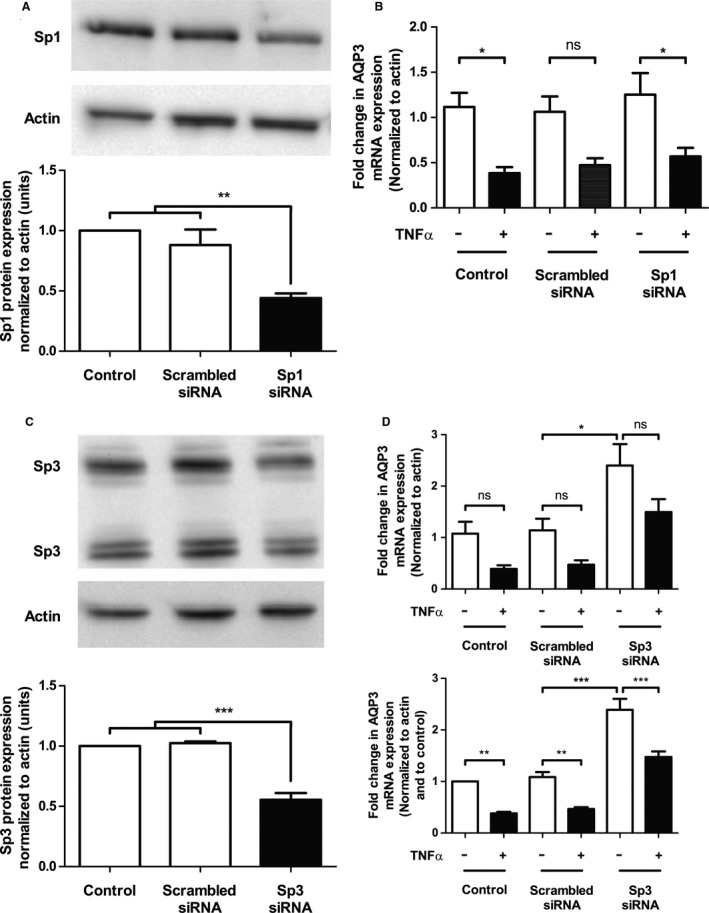
Knockdown of Sp3 increases constitutive AQP3 mRNA expression and partially blocks the TNF *α*‐induced decrease in AQP3 expression, whereas Sp1 knockdown does not alter AQP3 expression dynamics. Sp1 (A) and Sp3 (C) protein expression was assessed by western blot at 72 h following transient transfection with either 300 nmol/L scrambled or Sp1/Sp3 siRNA. Control cells were left untransfected. A representative blot is shown above each graph. Densitometry was performed on western blots (lower graph) and a ratio of Sp1 or Sp3 to actin normalized to the untransfected control was plotted, with statistical significance assessed by ANOVA and a Tukey's post hoc test. (*n* = 4; **P < 0.01; ***P < 0.001). (D) HT‐29 cells were transfected with 300 nmol/L scrambled or Sp1 (B)/Sp3 siRNA, followed by treatment with TNF *α* (25 ng/mL) for 12 h and assessment of AQP3 mRNA expression by real‐time RT‐PCR normalized to actin (top panel), and normalized to control (bottom panel) to account for intra‐assay variation. Control HT‐29 cells were left untransfected and statistical significance was assessed by ANOVA and a Tukey's post hoc test (*n* = 6; *P < 0.05).

In order to understand the effects of TNF*α* on AQP3 promoter activity, we generated a luciferase reporter construct containing all of the previously reported transcription factor sites characterized for the promoter region upstream of the AQP3 gene (Inase et al. 1995; Higuchi et al. 2007; Okahira et al. 2008). Luciferase expression was monitored in the absence of a transfection/transcription normalization control as we found that cytokine treatment altered expression of commonly available control constructs (data not shown). Luciferase expression was instead normalized in a given experiment to cells expressing the full‐length AQP3 promoter construct (Fig. 2). Importantly, TNF*α* did not significantly alter the baseline expression of luciferase in the pGL4.10 vector control lacking a promoter. Inclusion of the AQP3 promoter significantly increased luciferase expression and 12 h treatment with TNF*α* was able to downregulate the expression of luciferase in the full‐length (−1680), promoter truncation (−761, −346) and a site‐directed mutant construct (−346 lacking an Sp1 site) (Fig. 3). Furthermore, a distal truncation of the full‐length promoter (−966) resulted in reduced luciferase expression, whereas shorter truncations (−346) resulted in upregulation of promoter activity, suggesting a potential transcriptional suppressor site located between these two points. Together, these data suggest that TNF*α* is inhibiting the constitutive transcriptional activity of the AQP3 promoter.

### Two Sp1 transcription factor sites are found in the proximal aquaporin 3 promoter and can be modulated by the transcription factor Sp3

The Sp1 transcription factor binding sites in the AQP3 promoter have been predicted (Inase et al. 1995), but their functional importance in regulating AQP3 expression has not been determined. We sought to understand the relative contribution of two possible transcription factors that can interact with these sites, namely, Sp1 and Sp3. Targeted siRNA partially downregulated Sp1 (Fig. 3A) and Sp3 protein expression (Fig. 3C) as assessed by western blot and densitometry. Sp1 knockdown did not significantly alter the baseline constitutive expression of AQP3 mRNA transcript, nor did it prevent the TNF*α*‐mediated downregulation of AQP3 expression (Fig. 3B), suggesting that the transcription factor Sp1 is not involved. In contrast, partial knockdown of Sp3 resulted in the upregulation of constitutive AQP3 mRNA expression and also partially blocked the TNF*α*‐induced decrease in AQP3 mRNA expression (Fig. 3D). These results suggest that Sp3 is involved in the tonic suppression of the AQP3 promoter and that the suppressive actions of this transcription factor may be further enhanced by TNF*α*.

### Sp3 is constitutively bound to the aquaporin 3 promoter and its level is not altered by treatment with TNFα

**Figure 4:**
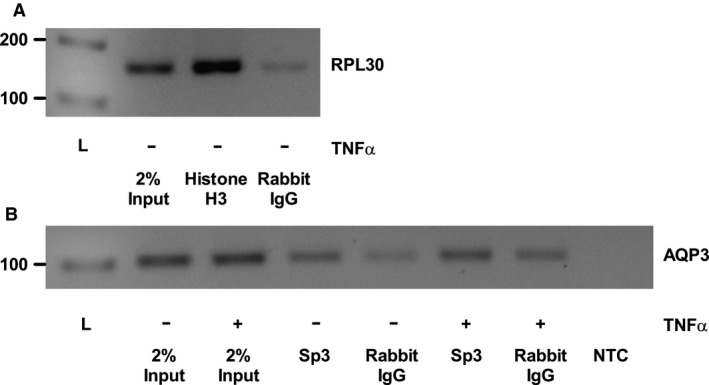
TNF *α* does not alter Sp3 binding to the AQP3 promoter in HT‐29 cells. Chromatin immunoprecipitation (ChIP) was performed on HT‐29 cells treated with TNF *α* (25 ng/mL) for 4 h. ChIP experimental success was verified using IP of Histone H3 bound to the Ribosomal protein L30 (RPL30, 160 bp) promoter in untreated samples (A). Sp3 antibody was used to IP chromatin, with an equivalent amount of rabbit IgG isotype antibody used as a negative control for nonspecific IP, and PCR was used to amplify the AQP3 promoter (AQP3, 101 bp) from the precipitate (B). Two percent of the total input into the ChIP reaction was used as a loading control to assess the equivalence of sample input, whereas a no template control (NTC) was used to ensure specific signal amplification in the PCR reaction. Relevant molecular weight markers from a 1 kb ladder (L) are shown on the left of the gels. A representative image for the ChIP results obtained is shown (*n* = 3).

Chromatin immunoprecipitation (ChIP) was used to both confirm the presence of the transcription factor Sp3 on the AQP3 promoter and to determine whether TNF*α* alters AQP3 promoter transcriptional activity through changes in transcription factor levels at the promoter. Success of the ChIP experiment was confirmed using positive control Histone H3‐binding enrichment to the RPL30 promoter relative to the enrichment observed with isotype control antibody (Fig. 4A). Similarly, Sp3 binding to the AQP3 promoter was enriched relative to IgG isotype ChIP in control cells, suggesting that Sp3 is constitutively bound to the AQP3 promoter. However, treatment with TNF*α* did not alter AQP3 promoter levels detected upon ChIP with the Sp3 antibody, and in combination with identical inputs into both ChIPs, the data suggest that Sp3 levels are not altered at the AQP3 promoter site as a potential mechanism driving the cytokine‐induced effect seen (Fig. 4B).

## Discussion

We found that TNF*α* was able to downregulate AQP3 mRNA expression in a time‐ and concentration‐dependent manner in HT‐29 cells. The inability to detect changes in AQP3 protein expression in HT‐29 cells using commercially available antibodies is in keeping with other findings in the literature, wherein upregulation of AQP3 at the mRNA level in human epithelial cell lines could not be similarly seen in western blot (Ben et al. 2008; Okahira et al. 2008). As TNF*α* was able to decrease tritiated glycerol uptake into HT‐29 cells, this finding supports the contention that changes in AQP3 mRNA expression resulted in concomitant functional changes at the protein level.

We found that the TNF*α*‐induced downregulation of AQP3 mRNA expression was mediated by both MEK/ERK signaling, as well as NF‐*κ*B signaling, results that paralleled the role of MEK/ERK in regulating AQP3 expression seen in several cell lines (Okahira et al. 2008; Horie et al. 2009; Huang et al. 2010; Wang et al. 2012). The luciferase reporter construct results suggest that a short proximal fragment of the AQP3 promoter is sufficient for constitutive transcription of this gene. These results are consistent with other AQP3 promoter studies, where various short fragments of the AQP3 promoter ranging from 60 to 420 bp were sufficient to drive transcription of luciferase (Ma et al. 1994; Inase et al. 1995; Higuchi et al. 2007). In addition, our data suggest that the TNF*α*‐mediated downregulation of AQP3 expression occurs through inhibition of constitutive transcriptional activity at the AQP3 promoter.

Transient transfection of HT‐29 cells with Sp3 siRNA resulted in the constitutive upregulation of AQP3 mRNA expression, a novel finding which indicates that Sp3 may be involved in the repression of the AQP3 promoter at baseline. Furthermore, decreased AQP3 mRNA expression induced by treatment with TNF*α* was partially abrogated in our studies, confirming that TNF*α* signals through Sp3 to mediate transcriptional inhibition of the AQP3 promoter. Similar experiments knocking down Sp1 expression did not have any effect on the baseline or TNF*α*‐mediated AQP3 transcription. ChIP studies revealed that Sp3 was bound constitutively to the AQP3 promoter in our system, likely allowing for TNF*α*‐mediated phosphorylation of this transcription factor so as to suppress AQP3 transcription further.

Future studies will be required to confirm our results in other cell model systems and in animal models of inflammatory disease where mucosal levels of TNF*α* are elevated. The HT29 cell line is a good system in that it responds to inflammatory cytokines in a similar matter as primary epithelial cells (Kolios et al. 1998). However, the HT29 clone that we used is nonpolarized, which may result in somewhat different cellular responses than a polarized epithelial cell monolayer would, although they do respond to TNF*α* with a downregulation of AQP3 expression as shown in other systems (Tancharoen et al. 2008; Horie et al. 2009). In addition, the HT29 cells were grown on plastic, which may also affect cellular responses differently than cells grown on a different substrate or on semipermeable filters.

Our findings are consistent with previous work suggesting that the presence of multiple GC boxes in a promoter tend to result in the inhibition of that promoter and transcriptional inactivity upon Sp3 recruitment, in contrast to promoters expressing a single site which are generally activated upon Sp3 binding (Suske 1999; Li et al. 2004). In the context of the AQP3 promoter, this possibility is plausible given that the promoter has been characterized in silico to possess 2 Sp1 binding sites (GC boxes) (Inase et al. 1995). It is also known that phosphorylation of the transcription factor Sp3 by p42/44 MAPK has the potential to alter its DNA‐binding and transcriptional abilities (Li et al. 2004), which may explain why the MEK inhibitor U0126 was capable of preventing the TNF*α*‐induced decrease in AQP3 mRNA expression. Given that Sp3 is involved in controlling the expression of numerous intestinal epithelial ion transporters, including NHE3 (Amin et al. 2006), the *γ*‐subunit of ENaC (Barmeyer et al. 2004) and sodium potassium ATPase (Markossian and Kreydiyyeh 2005), and is also responsive to TNF*α* (Amin et al. 2006), these striking regulatory similarities strongly suggest that changes in ion and water transport and their associated membrane channels are intricately linked. Our results characterize new transcriptional mechanisms controlling both constitutive and the TNF*α*‐mediated decrease in AQP3 expression, which when interpreted in the context of IBD and changes in ion and water transport associated with these diseases, helps to further our understanding of impaired barrier function.

## Conflict of Interest

The authors declare that they have no conflicts of interest with the contents of this article.
